# Questionable Utility of the Eccentric Utilization Ratio in Relation to the Performance of Volleyball Players

**DOI:** 10.3390/ijerph182211754

**Published:** 2021-11-09

**Authors:** Žiga Kozinc, Jernej Pleša, Nejc Šarabon

**Affiliations:** 1 Faculty of Health Sciences, University of Primorska, Polje 42, SI-6310 Izola, Slovenia; ziga.kozinc@fvz.upr.si (Ž.K.); 97200425@student.upr.si (J.P.); 2 Andrej Marušič Institute, University of Primorska, Muzejski trg 2, SI-6000 Koper, Slovenia; 3 Human Health Department, InnoRenew CoE, Livade 6, SI-6310 Izola, Slovenia; 4 Laboratory for Motor Control and Motor Behavior, S2P, Science to Practice, Ltd., Tehnološki Park 19, SI-1000 Ljubljana, Slovenia

**Keywords:** squat jump, stretch–shortening cycle, agility, vertical jump, 505 test

## Abstract

The difference between squat jump (SJ) and countermovement jump (CMJ), termed eccentric utilization ratio (EUR), is frequently suggested as an outcome that can be used in athletic training design. Unlike performance in SJ and CMJ, which is associated with sports performance, the association between EUR and sports performance is almost unexplored. This study aimed to investigate whether EUR is associated with performance in approach jump, linear sprint and change of direction (CoD) tasks in volleyball players. Forty-five male volleyball players performed SJ, CMJ, 25 m linear sprint, approach jump and two CoD tasks (505 test and modified T-test). EUR was calculated based on jump height, peak power, peak force and average power. SJ and CMJ variables showed moderate to high correlation with approach jump performance (*r* = 0.42–0.70), small correlation with modified T-test (*r* = 0.33–0.40) and small to moderate correlation with sprint time (*r* = 0.35–0.49). EUR showed only small associations with performance variables (*r* = 0.31–0.34). In all linear regression models with performance outcomes as dependent variables, the CMJ height was always the only statistically significant predictor. Our results support the recent arguments that the EUR offers limited insight into the neuromuscular capabilities of athletes.

## 1. Introduction

Vertical jumping tasks are frequently used for sports training [1] and to monitor athletes’ neuromuscular performance [2]. It is well documented that vertical jumping performance is associated with change of direction (CoD) ability [3] and linear sprinting performance [4,5]. While vertical jumping tests are useful to assess neuromuscular performance, the basic outcomes (e.g., jump height, peak power) offer only limited amount of information, which warrants a more detailed approach to testing. For instance, force–velocity profiling has been recognized as a promising tool to obtain a more comprehensive overview of an individual’s capability and their deficits and then use individually tailored exercise to optimize training [6]. Moreover, inter-limb asymmetries in jumping are known to impair sprint and CoD performance [7]. Finally, bilateral deficit in jumping tasks has been implicated as a possible factor for CoD performance [8]. These examples show that detailed analysis of vertical jumping can be useful to optimize the training for performance improvement. 

The squat jump (SJ) and the countermovement jump (CMJ) are among the most frequently used vertical jumps for assessment purposes [1,9]. On average, the height of the CMJ is slightly greater than the height of the SJ [9,10]. This difference between the jumps, often reported as the eccentric utilization ratio (EUR) (i.e., CMJ height divided by SJ height), has been suggested to serve as an indicator of performance [10]. Traditionally, it was believed that the difference between SJ and CMJ is largely determined by the capability to store the elastic energy during braking phase of the CMJ and use it during the propulsive phase [11]. In current strength and conditioning practice, EUR is used as an indicator of elastic storage in CMJ, which is a movement with the characteristics of a slow stretch–shortening cycle [12]. Lower values of EUR indicate that athlete should improve elasticity storage, which is addressed with explosive exercises with an emphasis on the transition part from eccentric to concentric muscle action (e.g., different variations of CMJs, Romanian rhythmic squats, hang clean, hang snatch and so on). On the other hand, athletes with higher values of EUR are usually directed toward training basic strength.

However, it was later shown that higher CMJ height can be, at least in most part, attributed to the simple fact that high forces in CMJ can be developed prior to the propulsive phase, which enables greater average power output in CMJ [13,14]. Thus, larger EUR could be explained by better ability to develop high forces in the downward phases of the CMJ. A recently published study presented a strong argument that higher EUR might not be beneficial at all [9]. In short, larger EUR can be a consequence of superior CMJ performance but may also lower SJ performance. Poor SJ performance could be related to poor ability to develop force rapidly [13,15] or to high levels of muscle slack [16]. This is supported by the fact that individuals with stiffer tendons, a trait that is beneficial for rapid force development, exhibit lower EUR [17]. Moreover, a recent study has shown that differences in EUR do not resemble differences in overall jumping ability across samples of athletes [18]. The study was performed on 712 male and female athletes from 9 different sport disciplines and 58 physical education students (total 770 participants). The major finding of this study was that the control group, comprising of physical education students, exhibited the highest EUR (approximately 19%), while track and field athletes, who showed the best overall jumping ability (SJ height = 39.5 cm, CMJ height = 43.6 cm), exhibited one of the lowest EURs among the tested groups (EUR was approximately 13%). Moreover, female athletes had slightly lower EUR compared to male athletes, with the same conclusion regarding the EUR and jumping ability. On the other hand, the only performance indicator in this study was CMJ and SJ height; thus, further research is needed to know more about associations between EUR and sport-specific performance.

Although EUR has been shown to be sensitive to training [10], its direct relationship with athletic performance has not been investigated. A study reporting improvements in EUR and jumping performance with training was conducted on a range of different athletes [10]. Athletes were tested two times, firstly in the off-season and secondly immediately prior to the start of the competitive season. Results of this study showed that jumping performance and EUR (+0.08–0.2) increased during this period, which could reflect that the increased amount of power training and SSC activities that are usually incorporated into the preseason training have a positive impact on EUR. On the other hand, improvements in jumping performance, with no change in EUR, have also been reported [19,20]. An 8-week interventional study (three groups; weightlifting, weight training and plyometrics) on untrained college-aged males reported that jumping height increased, while the EUR did not change significantly [19]. Furthermore, a 12-week interventional study (three groups; control, depth jump training, CMJ training) also reported increased jumping performance with no change in EUR [20]. Therefore, the utility of the EUR as performance indicator or guide for training design is still ambiguous. On the other hand, performance in SJ or CMJ has been reported to be positively associated with linear sprinting ability and CoD performance [21,22]. 

Moreover, the most frequently used assessment protocols for detecting the physical performance of volleyball players include different types of jumps, such as approach jump, block jump, which is similar to Sargent jump test but reaching with both hands (imitating volleyball block), and Abalakov jump test (CMJ with arm swing) [23,24]. Scientific literature also reports a wide use of change of direction assessment of volleyball players with the use of a modified T-test [25]. 

The purpose of this study was to investigate whether SJ and CMJ performances, and particularly the EUR, are associated with performance in approach jump, linear sprint and CoD tasks in volleyball players. According to the recent literature [9,18], we hypothesized that EUR will not be associated with selected performance tests. Volleyball was selected as the sport of interest, as it is characterized by versatile movements, involving jumps and brief explosive efforts in multiple directions [26,27].

## 2. Materials and Methods

### 2.1. Participants 

For this study, we recruited 45 young male volleyball players (age: 20.4 ± 3.4 years; body height: 186.5 ± 7.3 cm; body mass: 78.8 ± 7.4 kg). All the players had been competing in the 1st or 2nd division of the national league. They reported having been involved in regular training for 10.2 ± 4.1 years, attending 5.8 ± 1.8 training sessions per week, and regularly performing full body resistance exercises at least twice a week. All participants reported that they incurred no injuries in the previous 6 months. The participants were thoroughly informed about the experimental procedures and were requested to sign an informed consent before taking part in the experiment. For underage participants, their parents or legal guardians signed the consent on their behalf. The experiment was approved by the Republic of Slovenia National Medical Ethics Committee (approval no. 0120–99/2018/5) and was conducted in accordance with the Declaration of Helsinki.

### 2.2. Experimental Design

This was a cross-sectional study, with all measurements conducted in a single visit. The participants had been performing the testing procedures as part of their regular assessments. Thus, no familiarization session was conducted. The participants performed a standardized warm up, comprised of 10 min of light-intensity running, 5 min of dynamic stretching and 5 min of bodyweight resistance exercises. Then, they completed assessments of vertical jump on a force plate (SJ and CMJ) and performance tests (vertical approach jump, 25 m linear sprint, modified T-test and 505 test). The order of the performance tasks was randomized for all participants. 

### 2.3. Force Plate Jumps

The SJs and CMJs were performed on a piezoelectric force plate (Kistler, model 9260AA6, Winterthur, Switzerland). The participants performed two to three warm-up trials for each jump task. Then, each jump task was performed twice, with a 1 min break between trials. The hands were placed on the hips at all times. For the SJ, the participants were instructed to descend slowly to the initial position (which was determined when the knee angle was at 90°), stabilize and then perform the jump explosively, without any countermovement. In case the force signal dropped by more than 2% of the participant’s body mass, the countermovement was automatically detected by the software, and the trial was discarded and repeated. In addition, offline visual inspection of the force–time curves was performed, and the trials were discarded in case any countermovement was visually discriminable (<1% of cases). For the CMJ, the participants were instructed to start from the standing position and use an explosive countermovement (until the 90° knee flexion position) and to jump as high as possible. Ground reaction force data were recorded at sampling rate of 1000 Hz. The signals were immediately automatically processed by the manufacturer’s software (MARS, Kistler, Winterthur, Switzerland) by a moving average filter with a 5 ms window. The jump height, calculated based on take-off velocity, and peak force were considered as outcome variables. Furthermore, instantaneous power was calculated as the product of force and velocity, and the peak power and average power were considered as additional outcome variables. 

### 2.4. Vertical Jump with Approach

Vertical jumps with approach are often used in volleyball due to their resemblance to the spike jump [28]. Before the testing, the standing reach was measured with the dominant arm reaching overhead while the participants were facing the wall. Jumping reach was measured with measurement tape placed on the basketball board. Before each jump, participants chalked their fingertips to enable precise detection of the jumping reach. After an approach (the distance was self-selected), the participants jumped for height and touched as high as possible on the measure tape at the basketball board. All the participants were experienced volleyball players; thus, test familiarization was not needed. They were asked to perform the jumps in a manner that they found most convenient, similar to their personal technique during a volleyball practice. Each participant performed two warm-up trials at submaximal effort and three testing attempts, with 1 min breaks in between. Measurements were recorded to the nearest 1.0 cm. The difference between standing reach and jumping reach was calculated and taken for further analyses.

### 2.5. Change of Direction Performance

The change of direction assessment involved two tests (modified T-test and 505 test). For both tests, the single-beam laser timing gates (Brower Timing Systems, Draper, UT, USA) were used. The gates were positioned at hip level and recorded the times to the nearest 0.001 s. The participants began each task 30 cm behind the start line, to prevent early triggering. The modified T-test is very similar to traditional T-test but with approximately two times shorter total distance (total distance covered in modified T-test is 20 m, while in traditional T-test it is 36.5 m) (see Sassi et al. [29] for details). The participants started the test at their own will. Two warm-up repetitions with submaximal effort were performed first, followed by three test repetitions, with 2 min breaks in between.

For the 505 test, the participants were instructed to sprint to a line that was marked 15 m from the start line (with timing gates positioned 10 m form the start line) and plant the left or the right foot on the line, turn for 180° and sprint back 5 m through the timing gates again. Three attempts were performed for each leg in an alternating order, with 1 min breaks between the repetitions. In addition to raw 505 times, we calculated the CoD deficit, which is believed to represent a more isolated measure of CoD performance [30]. To obtain the CoD deficit, 0–10 m sprint times (see next section for details) were subtracted from the 505 test times. The 505 times and CoD deficits were averaged across the left and right leg before entering further analyses. 

### 2.6. Linear Sprint

Using five pairs of timing gates (the same as above), we collected 0–5, 0–10, 0–15 and 0–25 m sprint times. As in the sprint trials, the participants began each sprint 30 cm behind the start line. A standing start was used, and subjects were free to choose their front leg, which was kept constant across repetitions. Subjects were instructed to sprint from the start line through all sets of timing gates as fast as possible. Five trials were completed, and the breaks between the repetitions were set at 2 min. Sprint split times were used as performance indicators, and the 0–10 m time was also used for CoD deficit calculation (see previous section).

### 2.7. Statistical Analysis

Statistical analyses were done with SPSS (version 25.0, SPSS Inc., Chicago, IL, USA). Descriptive statistics are reported as mean ± standard deviation. The normality of the data distribution was verified with Shapiro–Wilk tests. The reliability was assessed with single-measures, two-way random model intra-class correlation coefficients (ICCs) for absolute agreement [31] and typical errors. Typical errors were divided by the mean values to obtain the coefficient of variation (CV). The reliability was determined to be acceptable when ICC was >0.75 and CV was <10%. One-way repeated measures analysis of variance with Bonferroni-corrected post hoc pairwise *t*-tests were conducted to check the differences in EUR across variables. The difference in corresponding variables in SJ and CMJ were checked with paired-sample *t*-tests. Correlations among SJ, CMJ and EUR variables and performance variables were assessed with Pearson’s correlation coefficients and interpreted as negligible (<0.1), weak (0.1–0.4), moderate (0.4–0.7), strong (0.7–0.9) and very strong (>0.9). Since a large number of correlation coefficients were calculated (96 in total), a Holm–Bonferroni sequential correction of *p*-values was applied to reduce the likelihood of Type 1 errors [32]. The correction was applied separately for the three blocks of correlation coefficients, with each block containing either SJ, CMJ or EUR variables, and all performance variables. Multiple linear stepwise regressions were done with performance variables as dependent variables and CMJ, SJ and EUR variables as candidate predictors. The successive predictors were included in the model if they statistically significantly (*p* < 0.05) contributed to the proportion of explained variance in performance variables. Durbin–Watson statistics and collinearity diagnostic tests were performed. We conservatively set the thresholds for the presence of collinearity at ≤0.3 for tolerance and ≥3 for variance inflation factor. The threshold for statistical significance was set at *p* < 0.05. 

## 3. Results

### 3.1. Reliability and Descriptive Statistics

The descriptive statistics for all variables is provided in Table 1. SJ and CMJ showed similar peak power (*t* = 1.2; *p* = 0.240). On the other hand, jump height, peak force and average power were significantly higher in CMJ (*t* = 4.1–20.3; all *p* < 0.001). The reliability was acceptable for all SJ variables (ICC = 0.86–0.88; CV = 3.8–8.2%), CMJ variables (ICC = 0.87–0.96; CV = 3.3–3.8%), approach jump (ICC = 0.99; CV = 2.94%), modified T-test (ICC = 0.91; CV = 2.9%), 505 test (ICC = 0.77; CV = 4.0%) and all sprint split times (ICC = 0.81–0.93; CV = 2.2–3.5%).

**Figure 1:**
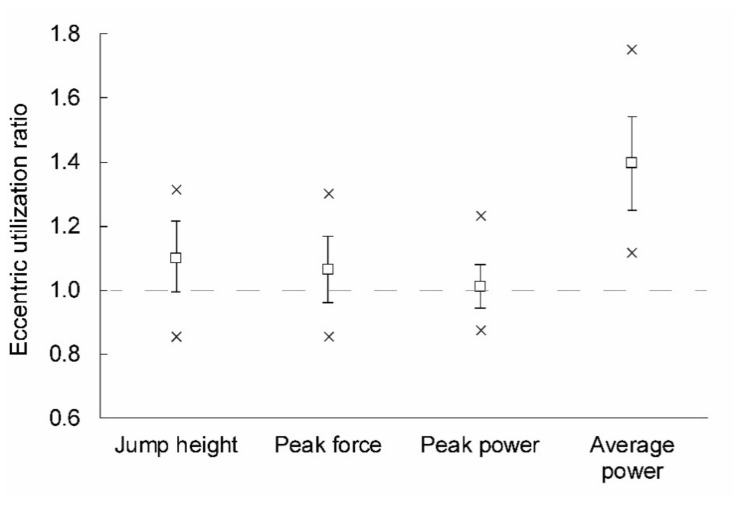
The eccentric utilization ratio across the outcome variables. Note that in addition to means and standard deviations, minimum and maximum values are depicted with a × symbol.

Figure 1 depicts how EUR behaved across variables. The analysis of variance showed that there were significant differences among variables in terms of the magnitude of the EUR (F = 151.68; *p* < 0.001). EUR in average power was the highest (1.40 ± 0.15) and was significantly different from the EUR calculated from the other variables (all *p* < 0.001). The EUR in peak power was the lowest (1.01 ± 0.07), and was also significantly different from the EUR calculated from the other variables (*p* = 0.001–0.024). EUR in jump height (1.11 ± 0.11) and peak force (1.07) were similar and not significantly different from each other (*p* = 0.692). 

### 3.2. Association between the Eccentric Utilization Ratio and Performance Outcomes

The approach jump height was in high positive correlation (all *p* < 0.01) with CMJ height (*r* = 0.74), peak power (*r* = 0.70) and average power (*r* = 0.70). In addition, moderate positive correlations were present between approach jump height and SJ height (*r* = 0.62), SJ peak and average power (*r* = 0.42–0.64) and CMJ peak force (*r* = 0.50). There was also a small correlation between approach jump height and EUR in average power (*r* = 0.34; *p* = 0.038). In linear regression model, CMJ height alone explained 53.6% of the variance in approach jump height, with no additional contribution of SJ or EUR variables. Modified T-test time showed small correlations (all *p* < 0.05) with SJ height (*r* = −0.36) and peak power (*r* = −0.33), all CMJ variables (*r* = −0.33 to −0.40) and EUR in peak force (*r* = 0.31). CMJ peak force explained 14.1 % of the variance in modified T-test times, with no additional contribution of SJ or EUR variables. Performance in the 505 test was correlated only to CMJ average power (*r* = −0.38). CoD deficit was not correlated to any SJ, CMJ or EUR variables (*r* = −0.14 to 0.26).

Performance in the 5 m sprint showed moderate correlation with CMJ height (*r* = −0.33), as well as with EUR in height (*r* = −0.31) and peak power (*r* = −0.31). CMJ height explained 9.1% of the variance in 5 m sprint time, with no additional contribution of SJ or EUR variables. The 10 m sprint time showed a small correlation with SJ peak power (*r* = -0.30; *p* = 0.046) and CMJ average power (*r* = −0.38; *p* = 0.010), as well as a moderate correlation (both *p* < 0.010) with CMJ height (*r* = −0.46) and CMJ peak power (*r* = −0.42). CMJ height explained 19.6% of the variance in 10 m sprint time, with no additional contribution of SJ or EUR variables. The 15 m sprint time showed a small correlation with CMJ peak power (*r* = −0.31) (both *p* < 0.05). CMJ peak power explained 8.1% of the variance in 15 m sprint time, with no additional contribution of SJ or EUR variables. The 25 m sprint time showed a small correlation with SJ average power (*r* = −0.35; *p* = 0.018) and a moderate correlation (all *p* < 0.01) with SJ height (*r* = −0.46), SJ peak power (*r* = −0.44) and all CMJ variables (*r* = −0.43 to −0.49). CMJ height explained 21.9% of the variance in the 25 m sprint time, with no additional contribution of SJ or EUR variables. No collinearity was detected in any of the regression analyses (all tolerance values >0.3, all variance inflation factors <3.0).

## 4. Discussion

The main purpose of this study was to examine whether EUR (i.e., the ratio between CMJ and SJ variables) is associated with linear sprint, approach jump and CoD performance. While some small correlations between EUR and performance variables were present, our results suggest limited utility of EUR for monitoring performance and designing training. In all regression models, CMJ height was included as the sole predictor of performance variables. Therefore, it seems that CMJ alone is likely better to monitor neuromuscular capacity in athletes, at least in relation to linear sprinting, approach jumping and CoD ability. 

The EUR, calculated as the ratio between SJ and CMJ, has been traditionally viewed as an indicator of the ability to utilize the stretch–shortening cycle [10] and was purported to be related in particular to the ability to store and reuse elastic energy [11]. However, strong arguments have been recently made that this might not be the case. In short, a large EUR could be a consequence of poor SJ performance, which can be related to poor ability of rapid force development and higher muscle slack [9]. Since a lower rate of force development and higher muscle slack are associated with poorer performance [16], EUR might not be a valid indicator of performance. It has been noted that EUR was larger in track and field athletes compared to that in gymnasts and parkour practitioners, while the jumping performance (SJ and CMJ) was better in gymnasts and parkour practitioners compared to that in track and field athletes [33]. The results of our study (correlations among basic jumps outcomes and EUR and performance measures) are presented in Table 2.

The limited practical utility of the EUR was also indicated in a recent study that showed that EUR is not the highest in groups of athletes who exhibit the best jumping ability [18]. Surprisingly, the control group of students showed higher EUR than all nine groups of athletes involved in the study. Considering the recent arguments regarding the EUR [9], we suggest that the control group of students could have presented poorer SJ performance, which could be due to poorer rapid force development and/or higher muscle slack. However, no indicators of performance were included in that study [18]; thus, no conclusion regarding the importance of EUR for sports performance could be drawn. In the present study, we confirmed our hypothesis, as EUR showed no or small correlations with performance indicators. This is also in accordance with recent evidence that the utilization of stored elastic energy plays very little role in CMJ, except when executed with low amplitude [34]. According to Schmidtbleicher [35], the CMJ is a slow stretch–shortening cycle task. Perhaps, tasks involving fast stretch–shortening cycles, such as drop jumps, would show higher associations with performance. One of the possible limitations of the EUR could be in the lack of a timing component. In contrast to EUR, reactive strength index, calculated as the ratio between drop jump height and contact time [12], and its modified version, calculated as the ratio between CMJ height and time to take-off [36], both involve a time component as well. The current literature suggests that the reactive strength index could be associated with CoD [37] but not linear sprinting performance [38,39,40]. Further research will clearly be needed to identify optimal performance monitoring indexes derived from vertical jumps. 

Our findings are also in accordance with previous interventional training studies. Gehri et al. [20] noted no increase in EUR after 12 weeks of plyometric training, while jumping ability was increased. Moreover, no statistically significant changes in EUR were noted after either weight lifting or plyometric training, despite significant increases in jumping ability [19]. Interestingly, Chelly et al. noted a higher increase in SJ height (7.1%) compared to the increase in CMJ height (4.2%) after 8 weeks of plyometric training [41]. It could be that the training contributed to tendon stiffness, which is a critical determinant of SJ performance [42]. Together with the results of the present study, the evidence suggests limited utility of the EUR in sport settings. Future interventional training studies should monitor EUR and possibly include baseline EUR as a covariate in their analysis to reveal whether there is any utility in this metrics at all. From a practical standpoint, the current evidence implies that coaches should probably not use EUR for decision-making regarding training design. 

Some limitations of the study with implications for future research should be acknowledged. The study sample was limited to male volleyball players; therefore, the results of the study cannot be generalized to female athletes and other sports. Moreover, the variables in the study covered only a limited aspect of performance. Other performance variables, particularly sport-specific variables (such as, for instance, ball serve or strike speed [43]), should be considered in future studies. Finally, our results must be interpreted with caution due to the cross-sectional study design. An interventional study (i.e., comparing training-based EUR with conventional training) would be needed to conclusively confirm that EUR has limited utility for sport-training design. 

## 5. Conclusions

In this study, we found that EUR showed very little association with CoD, approach jump and linear speed performance. While further studies are needed to conclusively confirm this, our results support the recent arguments that the EUR offers limited insight into the neuromuscular capabilities of athletes. Higher EUR might not even be desired, as it could be related to higher muscle slack and poor ability to develop force rapidly. This means that the use of EUR in practice should be reconsidered. 

## Figures and Tables

**Table 1:** Descriptive statistics for all outcome variables.

Outcome Variables	Mean	SD	Minimum	Maximum
SJ height (m)	0.39	0.06	0.26	0.53
SJ peak force (N/kg)	248.5	18.9	208.5	294.8
SJ peak power (W/kg)	57.61	6.17	42.12	72.82
SJ average power (W/kg)	23.18	3.25	18.40	31.13
CMJ height (m)	0.42	0.06	0.31	0.57
CMJ peak force (N/kg)	263.9	23.8	207.8	326.3
CMJ peak power (W/kg)	58.3	6.4	46.1	71.7
CMJ average power (W/kg)	32.2	4.2	24.2	43.7
EUR by height (m)	1.11	0.11	0.86	1.31
EUR by peak force (N/kg)	1.07	0.10	0.86	1.28
EUR by peak power (W/kg)	1.01	0.07	0.89	1.22
EUR by average power (W/kg)	1.40	0.15	1.11	1.76
Approach jump (cm)	77.1	9.4	53.7	94.7
Modified T-test (s)	5.37	0.25	4.89	6.00
505 test (s)	2.32	0.12	2.13	2.71
CoD deficit (s)	0.58	0.12	0.27	0.88
5 m sprint (s)	1.53	0.06	1.40	1.70
10 m sprint (s)	2.24	0.09	2.05	2.42
15 m sprint (s)	2.91	0.11	2.73	3.27
25 m sprint (s)	4.14	0.15	3.83	4.62

SD—standard deviation; SJ—squat jump; CMJ—countermovement jump; EUR—eccentric utilization ratio (CMJ/SJ); CoD—change of direction.

**Table 2:** Correlations among basic jump outcomes and eccentric utilization ratio and performance measures.

	Approach Jump	Modified T-Test	505 Test	CoD Deficit	Sprint 5 m	Sprint 10 m	Sprint 15 m	Sprint 25 m
SJ height (m)	0.62 **	−0.36 *	−0.17	0.08	−0.12	−0.30	−0.23	−0.46 **
SJ peak force (N/kg)	0.25	−0.05	0.01	0.14	−0.11	−0.15	0.05	−0.16
SJ peak power (W/kg)	0.64 **	−0.33 *	−0.10	0.16	−0.11	−0.30 *	−0.18	−0.44 **
SJ average power (W/kg)	0.42 *	−0.16	−0.29	−0.08	−0.08	−0.18	−0.04	−0.35 *
CMJ height (m)	0.74 **	−0.391 *	−0.13	0.26	−0.33 *	−0.46 **	−0.31	−0.49 **
CMJ peak force (N/kg)	0.50 **	−0.40 **	−0.27	−0.05	−0.11	−0.24	−0.17	−0.43 **
CMJ peak power (W/kg)	0.70 **	−0.37 *	−0.07	0.27	−0.28	−0.42 **	−0.32 *	−0.48 **
CMJ average power (W/kg)	0.70 **	−0.33 *	−0.38 *	−0.04	−0.030	−0.38 *	−0.24	−0.44 **
EUR by height (m)	0.02	0.00	0.09	0.24	−0.31 *	−0.21	−0.09	0.03
EUR by peak force (N/kg)	0.26	−0.31 *	−0.25	−0.14	−0.04	−0.11	−0.21	−0.28
EUR by peak power (W/kg)	0.06	−0.09	0.06	0.21	−0.31 *	−0.20	−0.22	−0.05
EUR by average power (W/kg)	0.34 *	−0.19	−0.11	0.08	−0.30	−0.26	−0.25	−0.09

CoDD—change of direction deficit; * *p* < 0.05; ** *p* < 0.01.

## Data Availability

The data are available upon request to the corresponding author.
